# High Concordance of Self-Reported and Objective Measures of Antihypertensive Use in Individuals Living with HIV in a Resource-Limited Setting

**DOI:** 10.5334/gh.1544

**Published:** 2026-03-26

**Authors:** Jane Jere, Julie T. Bidwell, Rita M. Butterfield, Allison Ruark, Torsten B. Neilands, Sheri D. Weiser, Nancy Mulauzi, James Mkandawire, Amy A. Conroy

**Affiliations:** 1 Invest in Knowledge, Zomba, Malawi; 2 Betty Irene Moore School of Nursing, University of California Davis, Sacramento, California, United States; 3 Center for AIDS Prevention Studies, University of California, San Francisco, San Francisco, CA, United States; 4 Health Professions, Wheaton College, Wheaton, IL, United States; 5 Division of HIV, Infectious Disease, and Global Medicine, University of California, CA, United States

**Keywords:** cardiovascular disease, hypertension, ASCVD risk score, resource-poor settings, sub-Saharan Africa, HIV/AIDS

In sub-Saharan Africa, people living with HIV are at increased risk for hypertension, and rates of controlled hypertension are extremely low (1). In resource-poor settings in sub-Saharan Africa, such as Malawi, medication availability is limited by supply and cost constraints (2,3), and patients often struggle to fill prescriptions (2). The absence of electronic health records in many health facilities further complicates the verification and tracking of patients’ use of antihypertensives. Self-reports of antihypertensive medication use can be susceptible to biases and inaccuracies, particularly in settings with low health literacy, and inaccurate self-reports could lead to suboptimal medical management. Objective data from medical records are typically considered more reliable, but in low-resource settings, medical records are not always available, necessitating alternative measures. In this study, we examined the reliability of self-reported antihypertensive medication use among individuals living with HIV and hypertension in Malawi.

Accurate reconciliation between patients’ prescribed and actual medication use is critical for clinical management and assessment of cardiovascular disease risk. Antihypertensive use is a key factor in the Atherosclerotic Cardiovascular Disease (ASCVD) risk score—a measure of 10-year risk for major cardiovascular events (e.g., nonfatal myocardial infarction, coronary heart disease, stroke) (4)—and thus directly influences risk scoring and classification (4). Although ASCVD risk scores are not routinely used in clinical care in Malawi, accurate reporting of antihypertensive medication use remains essential for cardiovascular risk assessment and research in limited-resource settings. Given the increasing prevalence of hypertension in Malawi (5), risk stratification, counselling, and initiation of pharmacological interventions is crucial for meeting the global targets of at least 50% of people receiving drug therapy and counselling to prevent heart attacks and strokes (6).

We examined data from the baseline visit of *Healthy Hearts*, a cohort study of couples living with HIV and cardiometabolic disorders (CMD) in Malawi. Details on *Healthy Hearts* are presented elsewhere (7). Couples were recruited from government and privately-funded HIV and CMD clinics in Zomba and Blantyre districts. Participants were ≥ 18 years, married/cohabitating for ≥ 6 months, and had at least one partner living with HIV and hypertension or diabetes (‘index patient’); a small proportion of participants were HIV-negative partners. Out of 500 individuals enrolled (250 couples), 233 participants with hypertension were included in this analysis. Ethical approval was obtained from the National Health Science Research Committee in Malawi and the Human Research Protection Program at the University of California, San Francisco. Participants provided written, informed consent.

To measure self-reported antihypertensive use, trained research assistants asked participants, ‘Are you currently taking any medications for your hypertension?’ (Yes/no). To generate a more objective measure of antihypertensive use, participants were asked to bring their portable medical record (‘health passport’) and all prescribed and over-the-counter medications (e.g., medication boxes, bottles, packets). Photographs were taken of health passport pages documenting prescription and medication packaging or containers, which captured medication names, dosage, and administration instructions. Photograph data was manually verified to create a complete list of current medications for each participant. The first author, J.J. (a Malawian medical doctor with clinical experience and training in research and public health) reviewed the list for any antihypertensive medications and created a variable (yes/no) indicating objective evidence of antihypertensive use. Objective evidence of antihypertensive use was defined as documentation of a prescription or antihypertensive use in the health passport and/or visual confirmation of medication container for antihypertensives.

We calculated the sensitivity and specificity of self-reported medication use to correctly identify individuals with objective evidence of antihypertensive use (sensitivity) or lack of use (specificity). We additionally calculated Cohen’s Kappa coefficient of agreement between self-reported and objective indicators. The Kappa coefficient accounts for observed agreement as well as agreement expected by chance, and has been previously used to assess agreement between self-reports and other objectively-measured indicators (8). We also captured medication adherence levels for antihypertensives using the Visual Analog Scale, which asks patients to indicate the proportion of the prescribed medication taken on a scale from 0–100% (9,10). All data management and statistical analyses were conducted using R version 4.4.2 (11).

Participants (N = 233) were 52.9 years of age on average (SD = 9.9); 49.4% were female and 70.1% had a primary school education or less. Nearly all (97.9%) participants were living with HIV, and 17.2% had diabetes. In terms of antihypertensive use, 82.8% (N = 193) self-reported current use, and 81.5% (N = 190) had an objective indicator of antihypertensive use. The distribution of self-reported and objective indicators of antihypertensive medication use is presented in Table 1. The sensitivity of the self-reported indicator was 98.4% and the specificity was 86.0%. Cohen’s Kappa coefficient of agreement between subjective and objective indicators was 0.867, indicating excellent or near-perfect agreement (12). Of the participants who self-reported current antihypertensive medication use, 140 (72.5%) reported perfect adherence (defined as 100%), 17 (8.8%) reported good adherence (defined as 80–99%), and 34 (17.6%) reported low adherence (defined as < 80%). Similar levels of adherence were found for the objective measure: of the participants who had objective evidence of antihypertensive use, **133 (69.3%) reported perfect adherence, 17 (8.8%) reported good adherence, and 35 (18.2%) reported low adherence**.

**Table 1:** Distribution of self-reported and objective indicators of antihypertensive medication use in Healthy Hearts participants with hypertension (N = 233).


*SELF-REPORTED ANTIHYPERTENSIVE USE*	*OBJECTIVE INDICATOR(S) OF ANTIHYPERTENSIVE USE*		TOTAL

	TAKING ANTIHYPERTENSIVES	NOT TAKING ANTIHYPERTENSIVES	

Taking Antihypertensives	187 (80.3%)	6 (2.6%)	193

Not Taking Antihypertensives	3 (1.3%)	37 (15.9%)	40

Total	190	43	233


*Note*: Cell percentages are proportions of the full sample. Self-reported medication use was collected by asking participants, ‘Are you currently taking any medications for your hypertension?’ (Yes/no). Objective indicators of antihypertensive use were collected through a review of medication containers brought to the study visit, as well as a review of the patient’s medical record (health passport).

Evaluating medication use through medical records is typically preferred over self-report. However, relying on records has limitations, especially in resource-limited settings where medical records are often incomplete or unavailable due to the lack of electronic health systems (13). Moreover, patients may not bring their medication containers or health passports to their clinical visits due to forgetfulness or logistical constraints. Self-report is faster and less resource-intensive to collect, but has been historically considered less reliable. Patients may forget medication names or dosages, particularly with complex regimens involving multiple drugs for the management of hypertension and other comorbidities, including HIV (14). Recall bias, social desirability bias, and misunderstandings about medication regimens can further undermine self-report reliability (14). These may be exacerbated in low-health-literacy settings, where patients may not fully comprehend the purpose or importance of their medications.

In this study, 98.4% of participants with objective evidence of current use correctly self-reported antihypertensive medication use, with a Kappa coefficient of 0.867 indicating a near-perfect agreement between self-reported and objective data. Our findings suggest that self-reports are highly reliable for identifying antihypertensive medication use in this population. Self-reported adherence was high and comparable between self-reported and objective measures (81.3% vs. 78.1%, respectively). This is a positive finding. Given that objective validation is a time-consuming and labour-intensive process, our findings have important implications for advancing hypertension research and clinical practice in resource-limited settings.

Several limitations are noted. While antihypertensive medication use can inform ASCVD risk scores, long-term adherence remains an important predictor of cardiovascular outcomes, especially in settings which do not routinely rely on risk scores for disease management. Additionally, it is plausible that concordance between self-reported and objective medication use is lower in government-funded clinics with unaffordable medications as compared to privately-funded clinics. Moreover, this study focused on people living with HIV who may have higher health literacy and greater access to care and thus may be more likely to produce reliable self-reported medication use. Nonetheless, our findings provide novel and valuable insights for similar limited-resource settings.
